# Buckyballs conjugated with nucleic acid sequences identifies microorganisms in live cell assays

**DOI:** 10.1186/s12951-017-0315-0

**Published:** 2017-11-09

**Authors:** Qingsu Cheng, Bahram Parvin

**Affiliations:** Department of Electrical and Biomedical Engineering, University of Nevada, Reno, NV 89557 USA

**Keywords:** C60, 16S rRNA, Microbial live cell assays

## Abstract

**Background:**

Rapid identification of bacteria can play an important role at the point of care, evaluating the health of the ecosystem, and discovering spatiotemporal distributions of a bacterial community. We introduce a method for rapid identification of bacteria in live cell assays based on cargo delivery of a nucleic acid sequence and demonstrate how a mixed culture can be differentiated using a simple microfluidic system.

**Methods:**

C60 Buckyballs are functionalized with nucleic acid sequences and a fluorescent reporter to show that a diversity of microorganisms can be detected and identified in live cell assays. The nucleic acid complexes include an RNA detector, targeting a species-specific sequence in the 16S rRNA, and a complementary DNA with an attached fluorescent reporter. As a result, each bacterium can be detected and visualized at a specific emission frequency through fluorescence microscopy.

**Results:**

The C60 probe complexes can detect and identify a diversity of microorganisms that include gram-position and negative bacteria, yeast, and fungi. More specifically, nucleic-acid probes are designed to identify mixed cultures of *Bacillus subtilis* and *Streptococcus sanguinis*, or *Bacillus subtilis* and *Pseudomonas aeruginosa.* The efficiency, cross talk, and accuracy for the C60 probe complexes are reported. Finally, to demonstrate that mixed cultures can be separated, a microfluidic system is designed that connects a single source-well to multiple sinks wells, where chemo-attractants are placed in the sink wells. The microfluidic system allows for differentiating a mixed culture.

**Conclusions:**

The technology allows profiling of bacteria composition, at a very low cost, for field studies and point of care.

**Electronic supplementary material:**

The online version of this article (10.1186/s12951-017-0315-0) contains supplementary material, which is available to authorized users.

## Background

Bacteria have long been the dominant form of life, their abundance is approximately 10^30^ prokaryotes on Earth [1], and they constitute the most significant proportion of the genetic diversity on the planet [2]. Microorganisms live, communicate, and interact with one another across time and space within their respective environmental substrates. For example, the soil microbiome plays an important role in all aspects of soil processes and the health of the ecosystem [2, 3], i.e., their influence on plant diversity and productivity [2]. Similarly, microorganisms play an important role in the health of human and animal. More specifically, (i) bacterial community in the rhizosphere influences plant growth (e.g., by supplying nitrogen, carbon) and response to pathogens [4], (ii) perturbations in the gut microbiota have been linked to neurological disorders [5], and (iii) presence and abundance of specific bacteria in the oral microbiome has been linked to different types of cancer [6–8]. In the first case, *Pseudomonas aeruginosa* and *Bacillus subtilis* are not only involved in the plant growth, but also form a symbiotic relationship with the plant root by forming biofilms that protect the plant, caused by infections bacteria [9, 10], and, at the same time, receiving nutrients from the plant. In the latter cases, *Helicobacter pylori* infection, found in the oral cavity [6], has been associated with gastric adenocarcinoma [11].

Because of these observations and the emerging significance of microorganisms in the health of human and ecosystem, identification of bacteria is necessitated. Although the 16S rRNA sequencing technology has provided insights into the presence and abundance of bacteria in a given model system, and, more importantly, identified biomarkers between the controlled and a perturbed system, sequencing is not the ideal technology for field or translational studies. Our approach focuses on identifying known and sequenced bacteria, has a very low cost, is rapid, applies to the field and translation studies, and can be scaled-up through multiplexing strategies.

Profiling microbiome for identifying and enumerating microorganisms has increasingly become routine in recent years [12, 13]. This is in part due to the PCR-based amplification of the bacterial 16S ribosomal RNA (rRNA) [13, 14] and proliferation of the Ribosomal databases that allows primers to be assessed and unique regions of 16S rRNA to be identified. Presently, characterizing spatial organization of microorganisms is limited to FISH-based technology in fixed assays through combinatorial labeling and spectral imaging [15]. Furthermore, bacteria are typically too small (e.g., the order of 1 um) to be recognized morphometrically using optical microscopy and different bacteria can share the same morphometry and shape features. If microbial identification can be performed in live cell assays then (i) the dynamics of the microbial organization can also be monitored as a function of the environmental parameters, and (ii) bacterial abundance and presence can be quantified, at a very low cost, by specially designed microfluidic systems. However, in live microbial assays, cargo delivery needs to overcome barriers such as the lipid membranes as well as the cell wall to target a specific nucleic acid sequence. In our earlier research [16], we demonstrated that functionalized Buckyballs are internalized within the cytosol and are non-sticky to the substrate. Although graphene-based materials have been proposed as a biosensor for detecting mutant DNA [17, 18] outside of cells, their applications for cargo delivery has been limited. We now leverage our previous results to show that Buckyballs (C60) conjugated with specific nucleic acid sequences can identify bacteria in live cell assays.

Alternative methods for molecular cargo delivery include guanidinium-rich molecular transporters (GR-MoTrs), polymer-based nanoparticles, and charged polymeric vectors. GR-MoTrs is a class of cell penetrating peptides (CPP) [19], which have been shown to internalize in several strains of algae, by crossing both the cell wall and the lipid membrane. However, these molecular transporters tend to have a more complex chemistry for cargo delivery and are sticky to the matrix substrate. Stickiness can be caused by a number of factors (e.g., charge distribution, hydrophobicity), and is quite important for a number of applications. Lipofectamine is a polymer-based nanoparticle and is another method for cargo delivery, but it is also known to be sticky to the natural environment [20]. In contrast, Buckyballs have a simpler chemistry for attaching cargo and are non-sticky to the substrate [16]. Finally, charged polymeric vectors (i) have a much higher molecular weight and of the order of 100 nm [21, 22], (ii) lower transfection efficiency of the order 15–50% [22–24], and (iii) can be sticky to the substrate. In contrast, C60 has a lower molecular weight, are of the order of 1 nm, and have higher efficiency as shown later.

The mechanism of internalization of carbon-based nanoparticles is not well understood for mammalian cells and even rarely studied for bacteria. Uptake of the nanoparticles in mammalian cells is either due to diffusion, endocytosis, or phagocytosis, where the latter is limited to a specific class of mammalian cells. Interestingly, while mammalian cells can uptake macromolecules through endocytosis, the process of an “endocytosis-like” phenomena has only recently been suggested for a specific class of bacteria [25]. This class of bacteria has a membrane-bound nucleoid that is similar to the eukaryotic nucleus [26]. Hence, we suggest that a viable transport mechanism for internalization of C60, in bacteria, is through the diffusion process. One possible control experiment would be a higher uptake in the presence of defects on the cell wall, which has been demonstrated in our earlier research [16].

In this paper, we show that functionalized C60s (i) label and detect a wide spectrum of microorganisms, (ii) distinguish two pairs of microorganisms in a live cell assay, (iii) can be integrated with a microfluidic system to differentiate a mixed culture. The mixed cultures include *B. subtilis* and *S. sanguinis,* and *B. subtilis* and *P. aeruginosa.* The first pair is a control experiment because these two species do not typically co-exist together. On the other hand, the second pair can co-exist together, *P. aeruginosa* has a low permeability in its outer membrane that may introduce new challenges, and the technology for rapid monitoring of these two species can be utilized for in situ bioremediation [27, 28] and plant growth [29, 30].

## Methods

### Synthesis of C60-RNA detector-DNA reporter complex and C60-fBSA

The oligonucleotides were purchased from Eurofins Genomics, and are shown below:

*Bacillus subtilis* 16S rRNA signature [31]: 5′-GAA GUC GUG AGG UAA CCU -3′.

*B. subtilis* 16S rRNA detector: 5′-AGG UUA CCU CAC GAC UUC AAA AA-AminoC7-3′, shown in Additional file 1: Figure 1a.

*B. subtilis* wrong 16S rRNA detector: 5′-AGG UUA CCU UUU GAC UUC AAA AA-AminoC7-3′, shown in.

*B. subtilis* reporter: 5′-Cy3-TGA AGT CGT GAG-3′, 5′-FAM-TGA AGT CGT GAG-3′. *S. sanguinis* 16S rRNA detector [32]: 5′-UAG CCG UCC CUU UCU GGU AAA AA-AminoC7-3′, shown in Additional file 1: Figure 1b.

*S. sanguinis* 16S rRNA wrong detector: 5′-UAG CCG UUU CUU UCU GGU AAA AA-AminoC7-3′. *S. sanguinis* reporter: 5′-5-FAM-TAC CAG AAA GGG-3′.

*P. aeruginosa* 16S rRNA detector [33]: 5′-GGU AAC CGU CCC CCU UGC AAA AA-AminoC7-3′, shown in Additional file 1: Figure 1c.

*P. aeruginosa* wrong 16S rRNA detector: 5′- GGT AAT UUU CCC CCU UGC AAA AA-AminoC7-3′.

*P. aeruginosa* reporter: 5′-Cy3-TAC ATG GAG GTC-3′.

To synthesize the detector and reporter C60 conjugates, the rRNA detector was first conjugated with C60-pyrrolidine tris acid (Sigma) as follows:

(I). The C60-pyrrolidine tris acid (1 nmole) was dispersed in 0.5 mL of 2-(N-morpholino) ethanosulfonic acid (pH 5.6) (MES) (Sigma) buffer under sonication for 30 min at ambient conditions.
(II). 0.25 mL of 1-(3-dimethylaminopropyl)-3-ethylcarbodiimide hydrochloride (0.4 mol/L) (EDC) (Sigma) and 0.25 ml of N-hydroxysuccinimide (0.2 mol/L) (NHS) (Sigma) in MES solution were added to activate the carboxylate groups [34, 35].
(III). The mixture was centrifuged at 12,000*g* for 30 min in a 5 kDa molecular weight cutoff centrifugal filter (Millipore) for five times to remove the excessive EDC and NHS and re-dispersed in MES buffer.
(IV). 5 nmole of rRNA detector was added into the c60-pyrrolidine tris acid solution at 4 °C overnight for conjugation.
(V). The final mixture was purified by centrifugation [36]. The stock concentration was set at 1 mg/mL.

Next, reporters (5 nmole) were hybridized on the purified rRNA detector-C60 in PBS. The reporters were added into the rRNA detector-C60 solution, and the mixture was heated to 75 °C and slowly cooled down for 5 h to allow hybridization. After hybridization, the reporter-rRNA detector-C60 probes were purified by centrifugation.

The protocol for fabrication of C60-fBSA is as follows:

(I). The C60-pyrrolidine tris acid (1 nmole) was dispersed in 0.5 mL of 2-(N-morpholino) ethanosulfonic acid (pH 5.6) (MES) (Sigma) buffer under sonication for 30 min at ambient conditions.
(II). 0.25 mL of 1-(3-dimethylaminopropyl)-3-ethylcarbodiimide hydrochloride (0.4 mol/L) (EDC) (Sigma) and 0.25 mL of N-hydroxysuccinimide (0.2 mol/L) (NHS) (Sigma) in MES solution were added to activate the carboxylate groups [34, 35].
(III). The mixture was centrifuged at 12,000*g* for 30 min in a 5 kDa molecular weight cutoff centrifugal filter (Millipore) for five times to remove the excessive EDC and NHS and re-dispersed in MES buffer.
(IV). 5 mg of fluorescent bovine serum albumin (fBSA) (Sigma) was added into the c60-pyrrolidine tris acid solution in PBS at 4 °C overnight for conjugation.
(V). The final mixture was purified by centrifugation [36]. The stock concentration was set at 1 mg/mL.

### Fluorescent microscopy

To investigate whether probes could differentiate *B. subtilis* and *S. sanguinis*, both wide-field fluorescent microscopy and super-resolution reconstruction were used. The live bacteria were incubated with two probes (100 nM) (5′FAM for *S. sanguinis* and Cy3 for *B. subtilis*) for 60 min and followed by a PBS wash. After that, 5 µL of bacteria were placed on a slide. The specimen was mounted in glycerol and imaged by fluorescent microscopy. In addition, *B. subtilis* with no probes, *S. sanguinis* with no probes, *B. subtilis* with *S. sanguinis* with probes, *S. sanguinis* and *B. subtilis* with probes were set as negative controls. *B. subtilis* with *B. subtilis* probe, *S. sanguinis* with *S. sanguinis* probe were set as positive controls.

For wide-field fluorescent microscopy, EVOS FL Auto Imaging System equipped with an AMEP 4700 100 × oil objective (1.28 of NA and 0.21 mm of WD) and a 40 × Olympus water objective (0.8 of NA and 3.3 mm of WDof 3.3 mm) was used. The excitation lasers were set at 488 nm for 5-FAM and 568 nm for Cy3. All other imaging parameters were set constant for all specimens.

For super-resolution microscopy, the specimens were air dried before mounting with glycerol. The slides were then imaged with General Electric DeltaVision OMX super-resolution microscope. The microscope is equipped with two EVOLVE 512, EMCCD cooled cameras. Excitation is provided by DPSS lasers at 488 nm/100 mw and 568 nm/100 mw. The objective is Olympus UPLanSApo, with 100×/1.4 NA Oil PSF, and used GE immersion oil of 1.514 RI at room temperature. Two cooled (− 79 °C) camera are used for image acquisition. Both cameras image at the samples with pixel resolution of x = y = 0.0792 $\upmu$ and z = 0.125 $\upmu {\text{m}}$. The first camera captures the green fluorescence with a field of view of 512-by-512 pixels, EMCCD = 10 MHz, EMCCD gain = 300, and exposure = 20 ms. Excitation using 488 nm laser is set at 31.3%, and the emission filter of 528/48 nm is used to capture the green fluorescence. The second camera captures the red fluorescence with a field of view of 512-by-512 pixels, EMCCD mode 10 MHz, EMCCD gain = 300, and exposure = 20 ms. Excitation, using 568 nm laser, is set at 31.3%, and the emission filter of 609/37 nm is used to capture the red fluorescence. For 3D reconstruction, 29 slices were collected at 0.125 microns per slice, and images rendered.

### Probe efficiency, crosstalk, and accuracy

To determine the efficiency of the probes, the single strain culture specimens were incubated with probe complexes. The efficiency is computed by Eq. (1):

1 $$\text{Efficiency}\, \left( \% \right) = \frac{{\text{The}\,\text{number}\,\text{of}\,\text{cells }\,\text{lit}\,\text{up}\,\text{by}\,\text{fluorescence}}}{{\text{The}\,\text{total}\,\text{number}\,\text{of}\,\text{cells} }} \times 100\%$$

To determine the cross-talk of *B. subtilis* and *S. sanguinis* probe complexes, a mixture *B. subtilis* and *S. sanguinis* was incubated with a mixture of probe complexes. Cross-talk is computed as follows. 

2 $$\text{Cross }\,\text{talk }\left( \% \right) = \frac{{\text{The }\,\text{number}\,\text{of }\,\text{cells}\,\text{with }\,\text{orange }\,\text{fluorescence }\,\text{signal}}}{{\text{The}\,\text{total}\,\text{ number }\,\text{of }\,\text{cells}\,\text{with }\,\text{any}\,\text{fluorescence}\,\text{signal}}} \times 100\%$$

To determine the accuracy of the probes, *B. subtilis*/*S. sanguinis* was mixed with *E. coli* and incubated with their specific probes. After the bacteria had been dried, the specimens were gram stained, imaged by microscopy and counted. The accuracy is given by Eq. 3.

3 $$\text{Accuracy} \,\left( \% \right) = \frac{{\text{The }\,\text{number}\,\text{of}\,\text{cells }\,\text{showed}\,\text{both }\,\text{fluorescence}\,\text{and}\,\text{gram }\,\text{positive }\,\text{stain }}}{{\text{The}\,\text{number }\,\text{of}\,\text{cells }\,\text{showed}\,\text{fluorescence}}} \times 100\%$$

### Cell culture

*Escherichia coli* (Invitrogen C404003), *M. tuberculosis* (ATCC 27294)*, Z rouxii* (ATCC 24905), *P. aeruginosa* (ATCC 27583) and *B. subtilis* (ATCC 9466) were kept in LB Broth at 37 °C. *C. reinhardtii* (Chlamy.org CC124) was cultured in provided medium. All microorganisms were used when the OD_630_ is at 0.6.

### Preparation of microfluidics

Glass slides (Fisher Scientific) were washed with ethanol, dried with air, and exposed to 4 mW/cm^2^ UV light (UVP, LLC) for 2 h. The hydrogel precursor (0.5 mL) consists of 10% (v/v) 700 MW PEG diacrylate (PEG-DA) (Sigma) and 0.5% (v/v) 2-hydroxy-2-methylpropiophenone (Sigma), and is evenly distributed over the glass slides by a spin coater (SCK-200P). The slides were then placed under approximately 4 mW/cm^2^ UV light for 15 s under a mask to gel. The slides were then incubated in 50 mM triethylene glycol mono-mercaptoundecyl ether (Sigma) for 15 min, rinsed in 70% ethanol for 15 min and washed with DI water. During this process, the microfluidics is stored in a humid environment to avoid desiccation.

### Dynamic monitoring of microorganisms

A mixture of *B. subtilis* and *S. sanguinis* were incubated with their mixed probes for 30 min. After the incubation, 500 µL of the mixture was centrifuged to remove media and resuspended in 20 µL growth medium. 0.5 µL of FeCl_2_ and glucose (5 mM, in growth medium) were placed in on the sinks of the microfluidic slides and incubated for 15 min in a humid chamber. Next, 0.2 µL of the labeled microbial pool was placed on the source side of the microfluidic chamber. After 15 min, random fields of view of the sink wells were selected for imaging.

A mixture of *B. subtilis* and *P. aeruginosa* were incubated with their mixed probes for 30 min. After the incubation, 500 µL of the mixture was centrifuged to remove media and resuspended in 20 µL growth medium. 0.5 µL of KNO_3_ and glucose (5 mM, in growth medium) were placed in on the sinks of the microfluidic slides and incubated for 15 min in a humid chamber. Next, 0.2 µL of the labeled microbial pool was placed on the source side of the microfluidic chamber. After 15 min, random fields of view of the sink wells were selected for imaging.

## Results and discussion

### Buckyballs functionalized with a fluorescent reporter labels a diverse set of microorganisms

In a recent publication, we showed that Buckyballs are internalized within the cytosol, and when functionalized with a fluorescent or radiotracer reporters, bacteria can be detected [16]. Here, we first show that functionalized Buckyballs with a fluorescent reporter can also (i) be internalized within the cytosol, and (ii) detect a diverse set of species such as gram-positive and negative microorganisms, non-gram staining bacteria, and eukaryotic cells.

In a similar protocol to our previous research [16], *E. coli* and *B. subtilis* were incubated with C60-pyrrolidine tris acid functionalized with fluorescein. Samples were then sectioned with a microtome and imaged with electron microscopy to show that fluorescein labeled Buckyballs are internalized in both species, as shown in Additional file 1: Figure 2.

**Fig. 1:**
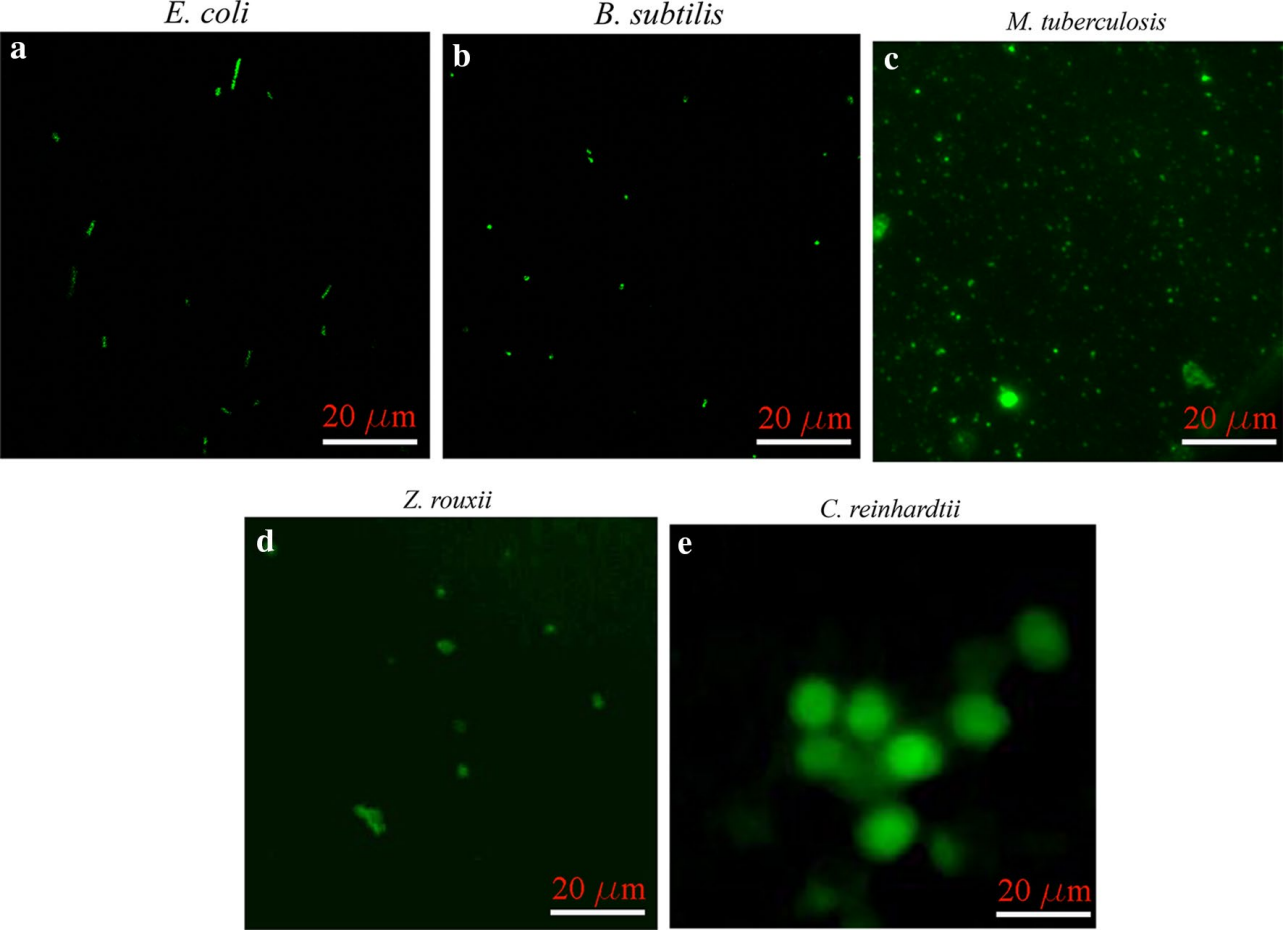
C60-fBSA can label both prokaryotes and eukaryotes microorganisms. Positive association of C60-fBSA with **a** gram-negative bacteria *E. coli*, **b** gram positive bacteria *B. subtilis*, **c** non-gram staining bacteria *M. tuberculosis*, **d** eukaryotic microorganisms *Z. rouxii*, and, **e** eukaryotic microorganisms *C. reinhardtii,* monitored with fluorescent microscopy. Scale bar is 20 µm

Gram-negative *E. coli* and Gram-positive *B. subtilis* were incubated with C60-fBSA for 30 min. Samples were then washed with the DI H_2_O to remove excess probes. Finally, live samples were imaged with confocal fluorescent microscopy. Figure 1a and b indicate a positive association of C60-fBSA with *E. coli* nor *B. subtilis* following excitation frequency by a 488 nm laser. The fluorescent emission is strictly due to C60-fBSA because neither *E. coli* nor *B. subtilis* have an auto-fluorescence signal under the same conditions. In addition, these fluorescent signals colocalize with *E. coli* and *B subtilis* cells in brightfield images, which suggests either internalization within the cell or binding to the cell wall [16].

*Mycobacterium tuberculosis* has a waxy coating, in terms of high lipid content, in its cell wall and does not retain traditional bacteriological stains. This bacterium can appear either as Gram-positive or negative. To investigate whether C60-fBSA can either attach themselves to the cell wall or be internalized, samples were subjected to the same protocol, i.e., incubation, wash, imaging. Figure 1c indicates a positive association of C60-fBSA with *M. tuberculosis* following excitation by laser at 488 nm. This observation indicates that cargo delivery with C60-fBSA is feasible in spite of the high acid fatty coating of *M. tuberculosis*.

To examine if C60-fBSA can label eukaryotic microorganisms, two model systems of *Z rouxii,* and *C. reinhardtii* were selected. *Z rouxii* is a genus of yeasts that is widely used in the food industry. *C. reinhardtii* is a single cell alga and is widely used for investigating photosynthesis and biofuel production. Samples of these two bacteria were prepared according to the previous protocol with the results shown in Fig. 1d and e, respectively. Because *C. reinhardtii* is of the order of 20 microns, optical sectioning clearly reveals that functionalized Buckyballs internalize within this microorganism.

### Buckyballs conjugated with nucleic acid sequences can identify bacteria

**Fig. 2:**
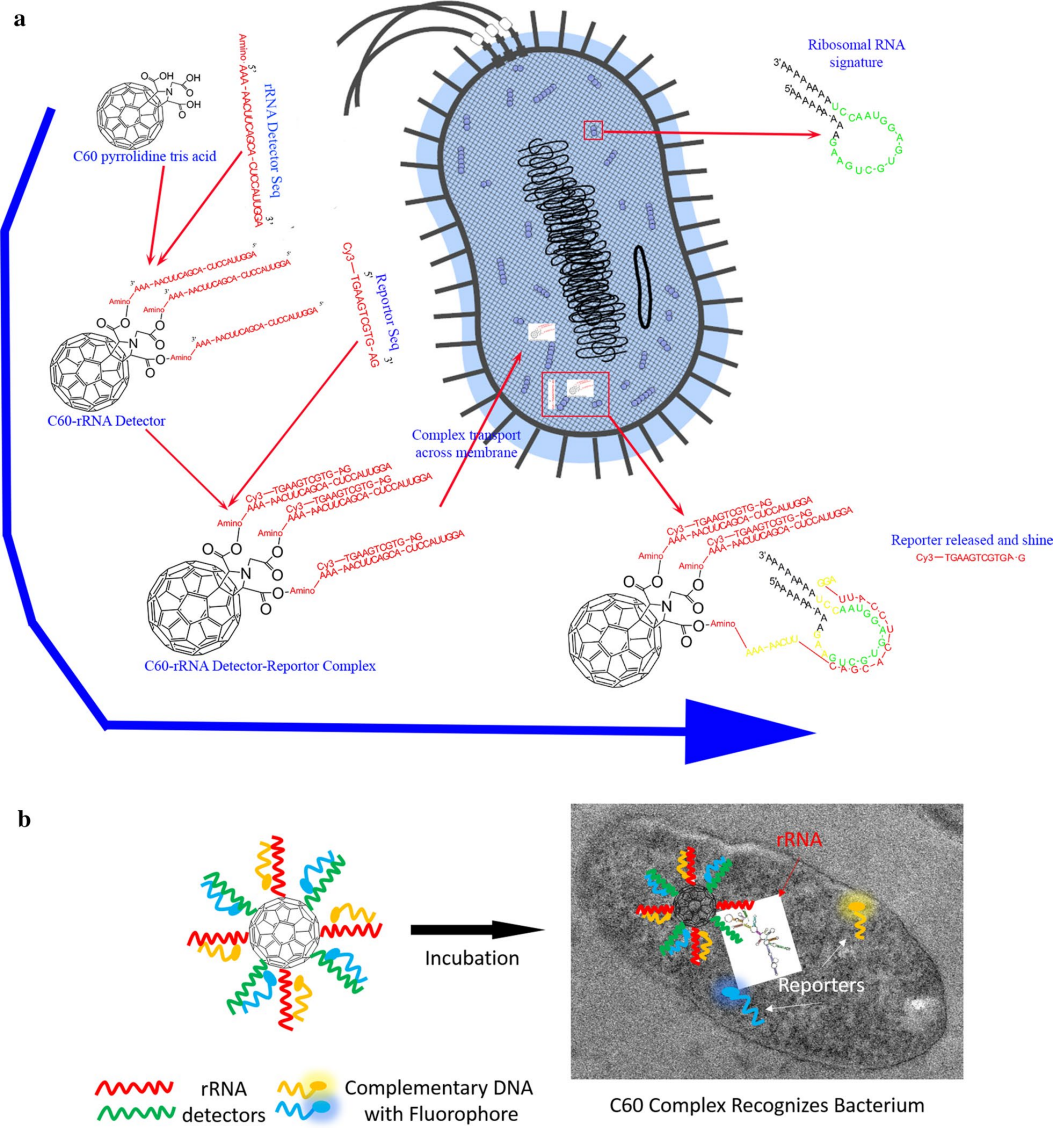
Schematic design for the C60-rRNA detector-reporter complex enables visualization of each microbe at a specific excitation frequency. The green and red reporters correspond to 488 and 568 nm excitation frequencies. **a** First, the C60 pyrrolidine is linked to an rRNA detector, which contains a complimentary sequence of a 16S rRNA region for a specific bacterium. Second, a DNA sequence with an attached fluorescence reporter is hybridized with the rRNA detector. At this point, the fluorescence signal is quenched. When the detector-reporter complex is incubated with the corresponding bacteria, and the C60-rRNA detector is recognized, the DNA reporter is released and fluoresces. **b** A brief cartoon describes how C60-rRNA detector-reporter complex enters the microbial cell, releases reporter and shines

We hypothesize that C60 pyrrolidine tris acid functionalized with a detector RNA oligo targeting a specific ribosomal RNA sequence can identify a unique microorganism. However, an additional nucleic acid sequence with a fluorescence reporter is needed for stability and imaging. The strategy to recognize a specific bacterium is shown in Fig. 2: (i) The C60 is functionalized with the RNA oligo that is complimentary to the bacterial rRNA sequence for a specific species. The specific rRNA sequences, for each bacterium, can be obtained from the published literature [15] or reference databases [37, 38]. (ii) The unstable RNA sequence is hybridized with complementary DNA sequence and an attached fluorophore reporter, where the fluorophore signal is quenched initially. (iii) The probe complex crosses the bacteria membrane and cell wall and releases the reporter if the bacteria contain the targeted ribosomal sequence. Subsequently, the released reporter fluoresces.

To validate the above hypothesis, C60-rRNA detector-reporter complexes were synthesized for simultaneous differentiation of *B. subtilis* and *S. sanguinis*. *B. subtilis* and *S. sanguinis* are both Gram-positive, and can not be differentiated with the gram staining. *B. subtilis* is involved in plant growth promotion and disease control [29], oil biodegradation efficiency [27], and biofilm formation for corrosion control [39, 40]. On the other hand, *S. sanguinis* is involved in periodontal diseases [41]. As an initial pilot project, it is prudent to differentiate two species that do not commonly live together. First, four control experiments were performed to visualize and validate the efficacy of C60-rRNA detector-reporter with nucleic acid sequences. (i) Neither C60 pyrrolidine tris acid nor C60-rRNA detector-reporter complexes showed any autofluorescence signal under 488 and 568 nm excitation laser, as shown in Additional file 1: Figure 3. (ii) An rRNA detector with three incorrect bases was synthesized to profile potential hybridization. However, none of these detector complexes were able to identify their target bacteria, i.e., *S. sanguinis* and *B. subtilis*. (iii) C60-rRNA detector-reporter complexes with correct sequences were incubated with non-targeted microorganisms to profile potential cross-talks, but no fluorescent signals were observed as shown in Additional file 1: Figure 4. (iv) The RNA-DNA-reporter complex, without being conjugated with C60, was incubated with the target bacteria. However, no fluorescence signal was observed, which suggests that conjugation with Buckyballs is a necessary step for nucleic acid cargo delivery. The RNA-DNA-reporter complex can also be replaced with DNA–DNA-reporter complex; however, our design has been influenced by the RNA FISH probes.

**Fig. 3:**
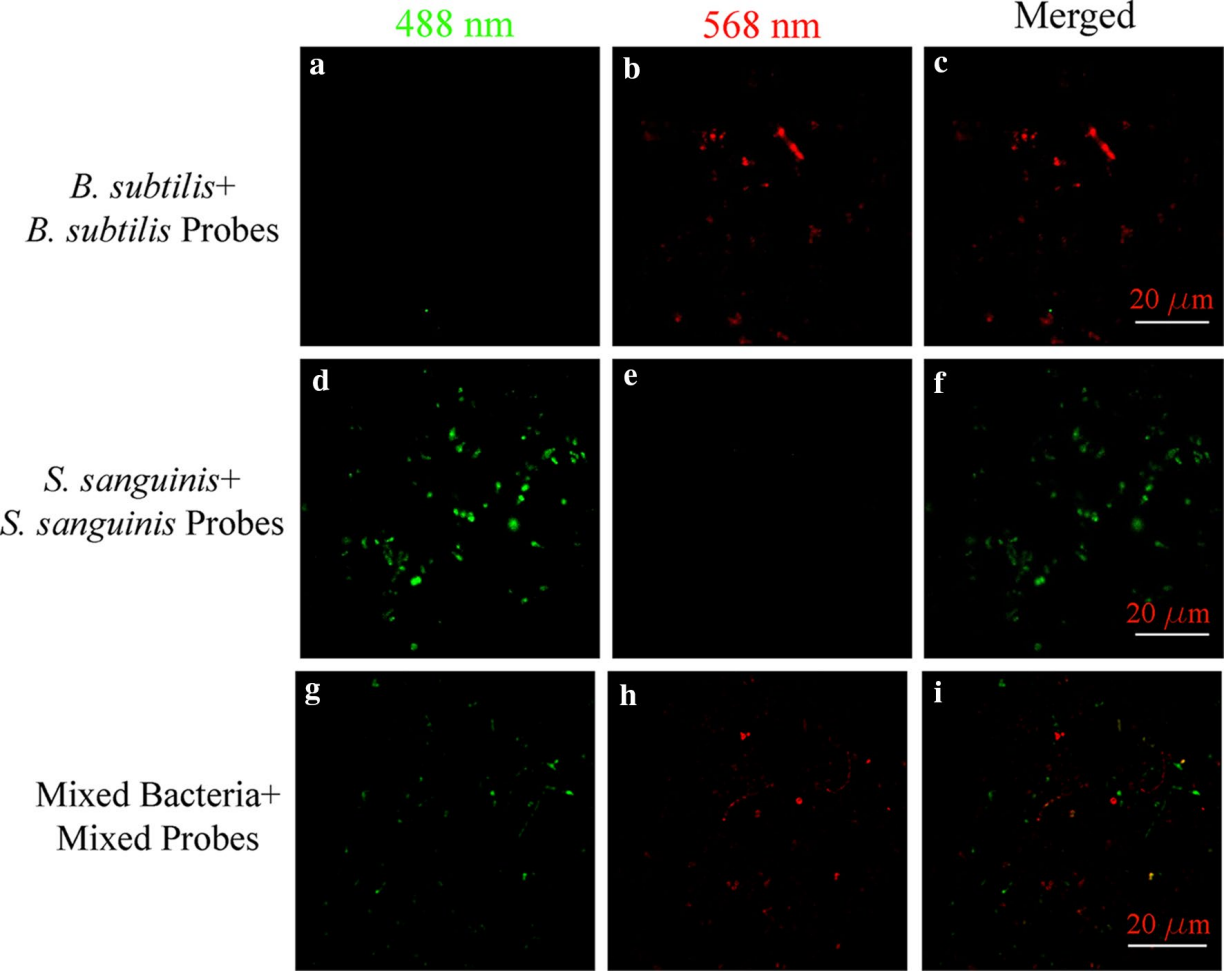
Hybridizations of two C60-RNA-DNA-reporter complexes with their corresponding bacteria, *B. subtilis* and *S. sanguinis,* are monitored by super-resolution microscopy. The reporters for *B. subtilis* and *S.* s*anguinis* are excited at 568 and 488 nm, respectively. **a-c** are *B. subtilis* incubated with *B. subtilis* probe complex. **a** shows no fluorescent signal when excited at 488 nm, **b** is the fluorescent signal at the 568 nm excitation frequency. **d-f** are *S. sanguinis,* incubated with *S. sanguinis* probe complex, **d** is fluoresces at the 488 nm excitation frequency, and **e** shows no fluorescence signal at the 568 nm excitation frequency. **g-i** are mixed bacteria treated with mixed probe complexes to indicate that each bacterium can be visualized at its corresponding excitation frequency

Having profiled and validated the synthesized sequences against a series of controlled experiments, two additional experiments were performed for simultaneous differentiation of *S. sanguinis* and *B. subtilis* in a live cell assay: (i) identifying each target microorganism *separately* with their specific sequence, and (ii) identifying a synthetic microbiome containing both microorganisms simultaneously with mixed synthesized sequences. Both of these experiments were monitored with super-resolution microscopy with a 50 nm spatial resolution [42, 43] with the results shown in Fig. 3. Although super-resolution microscopy is not a requirement, the intent was to examine if any ultrastructure can be elucidated. The results indicate that (a) *B. subtilis* and *S. sanguinis* have no autofluorescence under the excitation of 488 and 568 nm lasers; (b) C60-rRNA detector-reporter complex can differentiate and identify *B. subtilis* or *S. sanguinis* by their respective fluorescent emission; and (c) a mixture of *B. subtilis* and *S. sanguinis* probe complexes can distinguish composition of the two bacteria. A 3D reconstruction, from 29 serial sections, is shown in Additional file 2: Video 1. These experiments altogether confirm the fluorescent signal would be from the released reporter sequence only when the detector recognize the 16S rRNA signature inside bacteria. The field of view for this video was selected where maximum cross-talk occurs in the culture.

### C60 probes have low crosstalks

**Fig. 4:**
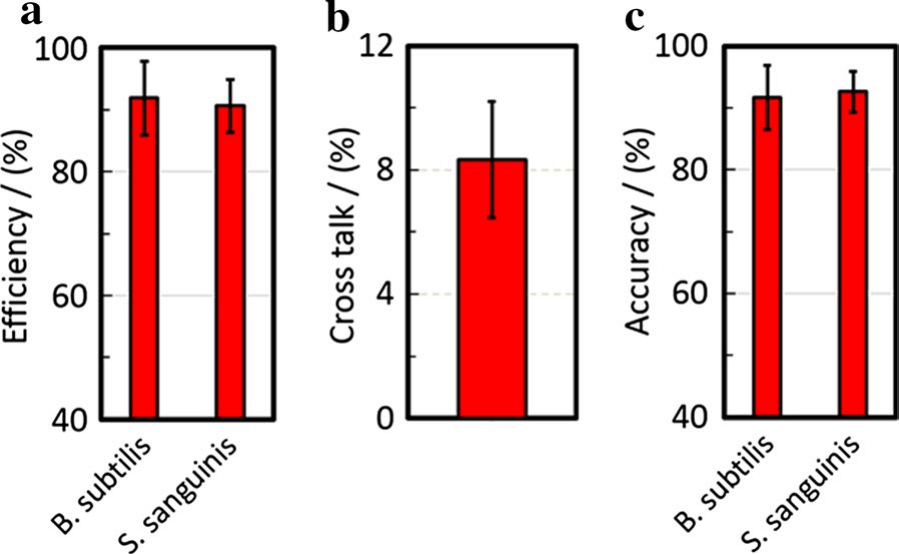
The probe efficiency, cross talk, and accuracy are reported. **a** The probe efficiency is more than 92%, indicating that more than 92% of bacteria is labeled in the mixed culture of *B. subtilis* and *S. sanguinis*. **b** The crosstalk is less than 8%, indicating that less than 8% bacteria are mislabeled within the labeled population of the coculture of *B. subtilis* and *S. sanguinis*. **c** The probe accuracy is greater than 92%, indicating that the probes can accurately label *B. subtilis* or *S. sanguinis* in the presence of *E. coli*

To further investigate the off-target effect of the C60-RNA detector-DNA reporter complex, the efficiency, cross talk, and accuracy were profiled. The efficiency is quantified as the ratio of cells that are fluorescent versus the total number of cells, where the total number of cells is counted by bright field microscopy. The cross talk is defined as the ratio of the number of cells that fluoresce in orange (e.g., co-localization of 488 and 568 nm probes), in a mixed culture (e.g., *S. sanguinis* with *B. subtilis*), versus the total number of fluorescently labeled cells. The accuracy is quantified by adding gram-negative bacteria, *E. coli,* to each of the mono-culture (e.g., *S. sanguinis* or *B. subtilis*), incubating the samples with their corresponding C60 probe-complexes, making slides, and then adding the gram-positive stain. The results are shown in Fig. 4. The RNA-DNA-reporter complexes can identify 92% of *S. sanguinis* with *B. subtilis* in all fields of views under observation. This 92% index is computed by manually counting colocalization of each microorganism in the fluorescence and bright field, simultaneously. Furthermore, with the fluorescently labeled population, only 8% of the bacteria demonstrated labeling with two fluorescent signals. Overall, the current design is slightly hindered by the crosstalk. However, the crosstalk can be further improved by adding an internal control (e.g., a secondary rRNA sequence) and/or refinement of the synthesis and purification process.

### Microfluidic chip validates differentiating of microorganisms

**Fig. 5:**
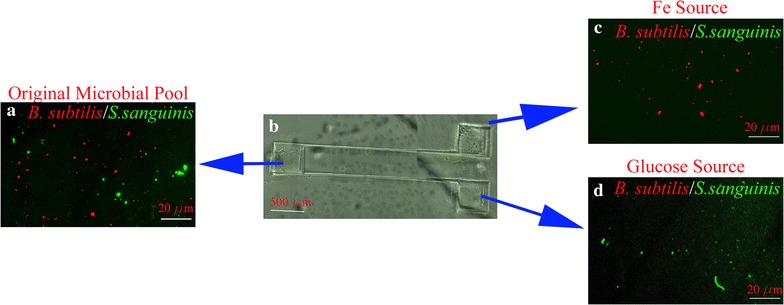
A mixed culture of labeled *B. subtilis* and *S. sanguinis* can be sorted, by their preferred chemoattractants placed in the sink wells of a microfluidic system. **a** *B. subtilis* and *S.sanguini*s were labeled with their corresponding probe complexes and placed in the well called the “original microbial pool.” **b** the original microbial pool, and Fe and glucose chemoattractants were placed in source and sink wells, respectively.  **c** *B. subtilis* migrated toward Fe^2+^ rich sink-well while, **d** *S. sanguinis* migrated to the glucose rich sink-well. The source and sink wells are monitored by fluorescent microscopy

**Fig. 6:**
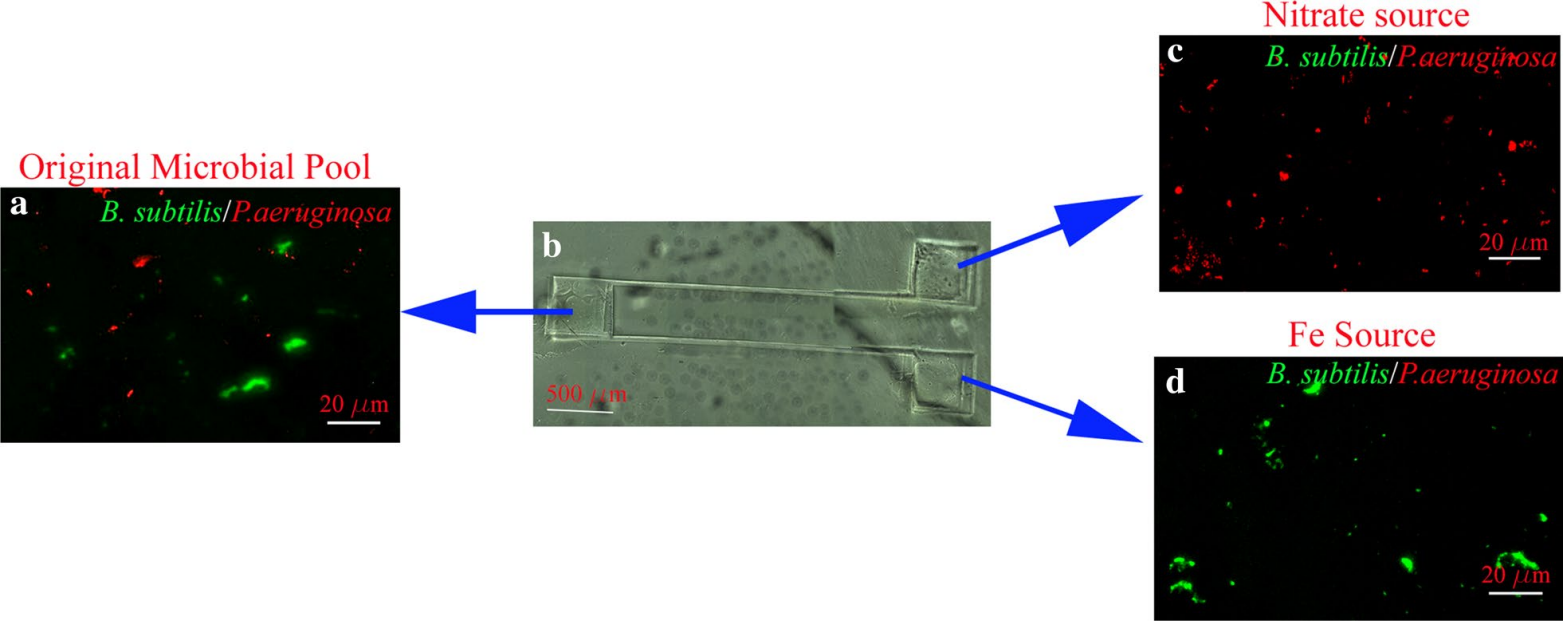
A mixed culture of labeled *B. subtilis* and *P. aeruginosa* can be sorted, by their preferred chemoattractants placed in the sink wells of a microfluidic system. **a** *B. subtilis* and *P. aeruginosa* were labeled with their corresponding probe complexes and placed in the well called the “original microbial pool.” **b** the original microbial pool, and nitrate and Fe chemoattractants were placed in source and sink wells, respectively. **c** *P. aeruginosa* migrated to the nitrate rich sink-well while **d** *B. subtilis* migrated toward Fe^2+^ rich sink-well. The source and sink wells are monitored by fluorescent microscopy

To demonstrate applications of C60-RNA-detector-DNA-reporter complex, a microfluidic system was designed, and two studies were performed for differentiating mixtures of bacteria using their respective chemoattractant. The microfluidic system is shown in Figs. 5b and 6b, which consists of one source well and two sink wells that are connected with channels. Labeled bacteria mixture were placed in the source well, and the corresponding chemoattractant media, for each bacterium, was placed in the sink wells.

In the first application, a mixture of labeled *S. sanguinis* with *B. subtilis* was placed in the source-sink (Fig. 5a) with chemoattractant, i.e., glucose (Fig. 5c) and FeCl_2_ (Fig. 5d) placed in the sink wells. After 30 min incubation, bacteria were differentiated into their preferred sink wells. It is estimated that less than 1% of the bacteria were directed into the wrong sinks. In the second application, the same microfluidic system was used to examine if a mixture of *B. subtilis* and *P. aeruginosa* (Fig. 6a), placed in the source well, can be differentiated by their corresponding chemo attractants of FeCl_2_ (Fig. 6d) and KNO_3_ (Fig. 6c), respectively. The experimental conditions were kept identical, and the sink wells were imaged after 30 min of incubation. The two bacteria were attracted to their preferred sinks, and it is estimated that less than 3% of the bacteria were directed into the wrong sinks. In contrast, when the media in the sink was replaced with PBS, no differentiation took place in either of the above two applications. The second application is an example of identifying and monitoring the presence of important soil bacteria in the rhizosphere. One of the rationales for selecting *P. aeruginosa* is that it has a low permeability in its outer membrane [44], and to demonstrate that our assay is valid for this class of microorganisms. The technique enables in situ sampling of the rhizosphere for rapid surveillance and monitoring bacteria species involved in the plant growth-promoting rhizobacteria as a function of environmental conditions [45, 46].

## Conclusion

This paper presented a comprehensive methodology for imaging microorganisms in live cell assays. We showed that functionalized Buckyballs could (i) detect a wide spectrum of microorganisms, and (ii) identify several species in live cell assays. The main design concept, for the probe complex, is to decorate Buckyballs with the nucleic acid probes that target a specific region of the ribosomal RNA for a specific microorganism. The synthesis has been validated for differentiating two pairs of bacteria. The RNA-DNA-reporter complex indicated a high level of detection rate, accuracy, and relatively low crosstalk. As a potential application, bacteria differentiation is demonstrated with a custom-designed microfluidic system. Such a system can be extended for assay development and optimization of bacterial culture conditions with potentially hundreds of sinks, which can lead to highly efficient multiplexed assays. The experimental configuration also allows for printing a microbe-specific RNA-DNA-reporter complex in each sink well and pushing (e.g., using a syringe) a microbial mixture, located in the source well, toward sinks, where hybridization will take place. Such a configuration and experimental setup will lead to highly multiplexed assays.

## Additional files

**Additional file 1.** Additional figures.

**Additional file 2.** 3D reconstruction was generated from 29 serial sections taken by super resolution microscropy, showing *B. subtilis* and *S. sanguinis* are distinguished by mixed probe complexes.

## Abbreviations

rRNA: ribosomal ribonucleic acid
PCR: polymerase chain reaction
FISH: fluorescence in situ hybridization
GR-MoTrs: guanidinium-rich molecular transporters
DNA: deoxyribonucleic acid
FAM: fluorescein
Cy3: cyanine 3
MES: 2-(N-morpholino)ethanosulfonic acid
EDC: 1-(3-dimethylaminopropyl)-3-ethylcarbodiimide hydrochloride
NHS: N-hydroxysuccinimide
PBS: phosphate buffered saline
WD: working distance
NA: numerical aperture
fBSA: fluorescent bovine serum albumin
