# Supplementary material for: Buckyballs conjugated with nucleic acid sequences identifies microorganisms in live cell assays
Source: J Nanobiotechnology. 2017 Nov 9;15:78. doi: 10.1186/s12951-017-0315-0 (PMC5679147; doi:10.1186/s12951-017-0315-0)
Supplement: Supplementary file 1 — Additional file 1. Additional figures. [file 12951_2017_315_MOESM1_ESM.docx]

**Additional File 1**

Buckyballs conjugated with nucleic acid sequences identifies microorganisms in live cell assays

*Qingsu Cheng and Bahram Parvin*

Department of Electrical and Biomedical Engineering

University of Nevada, Reno 89557, USA

**Additional Figure Captions**

| Additional Figure S1. Web server predicts the secondary structure of RNA detectors. (a) *B. subtilis* detector RNA shows hairpin structure for rRNA detection. (b) *S. sanguinis* detector RNA shows hairpin structure for rRNA detection. (c) *P. aeruginosa* detector RNA shows hairpin structure for rRNA detection. |
| --- |
| Additional Figure S2. Cellular uptake of C60- Fluorescein by *E. coli* and *B. subtilis* monitored by Transmission Electron Microscopy. The dark spots, pointed by the yellow arrows, represent C60-Fluorescein uptake by *E. coli* (a) and *B. subtilis* (b). Cellular uptake of C60 pyrrolidine tris-acid was reported in the previous publication [[1](#_ENREF_1)]. Overall, the results show that the functionalization of C60 pyrrolidine tris-acid does not affect diffusion of the C60 complex into bacteria cell body. |
| Additional Figure S3. Auto-fluorescence of the C60-rRNA detector-reporter complexes, synthesized for *B. subtilis* and *S. sanguinis* specific probes, is reported in the absence of the bacteria and monitored by fluorescent microscopy. (a-c) C60 pyrrolidine tris acid, C60-rRNA detector-reporter complex synthesized for *B. subtilis,* and C60-rRNA detector-reporter complex synthesized for *S. sanguinis* monitored in bright field. (d-f) auto-fluorescence of fields of views from (a-c) monitored by the fluorescence microscopy at the 488nm excitation frequency. (g-i) Auto-Fluorescence of fields of views from (a-c) monitored by the fluorescence microscopy at the 568nm excitation frequency. Scale bar is 10 µm. |
| Additional Figure S4. Mismatch incubation of probe complexes with bacteria shows no hybridization monitored by fluorescent microscopy. (a) *S. sanguinis* probe complexes incubated with *B. subtilis* bacteria imaged in bright field. (b-c) *S. sanguinis* probe complexes incubated *B. subtilis* have no fluorescent signal under 488 or 568nm excitations frequencies. (d) *B. subtilis* probe *complexes* incubated with *S.* *sanguinis* and imaged in the bright field. (e-f) *B. subtilis* probe complexes incubated with *S. sanguinis* have no fluorescent signal under 488 or 568nm excitations frequencies. Scale bar is 10 µm. |


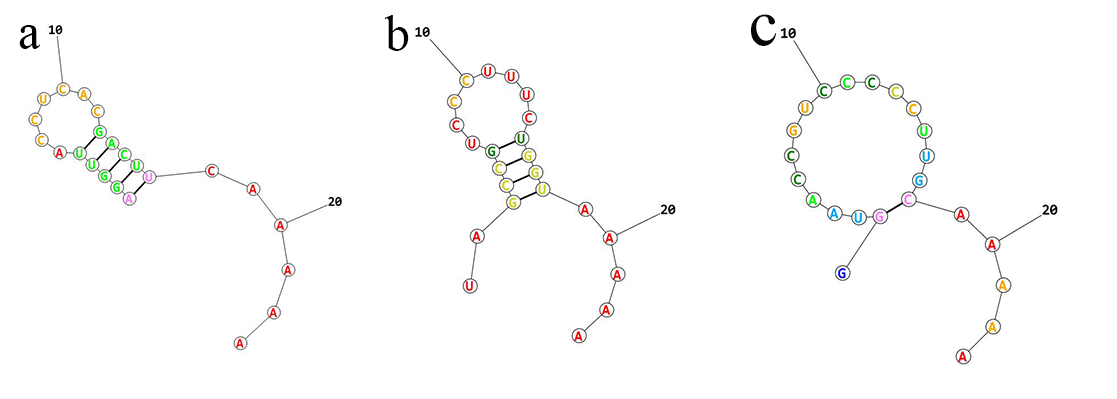


Additional Figure S1. Web server predicts the secondary structure of RNA detectors. a) *B. subtilis* detector RNA shows hairpin structure for rRNA detection. b) *S. sanguinis* detector RNA shows hairpin structure for rRNA detection. © *P. aeruginosa* detector RNA shows hairpin structure for rRNA detection.


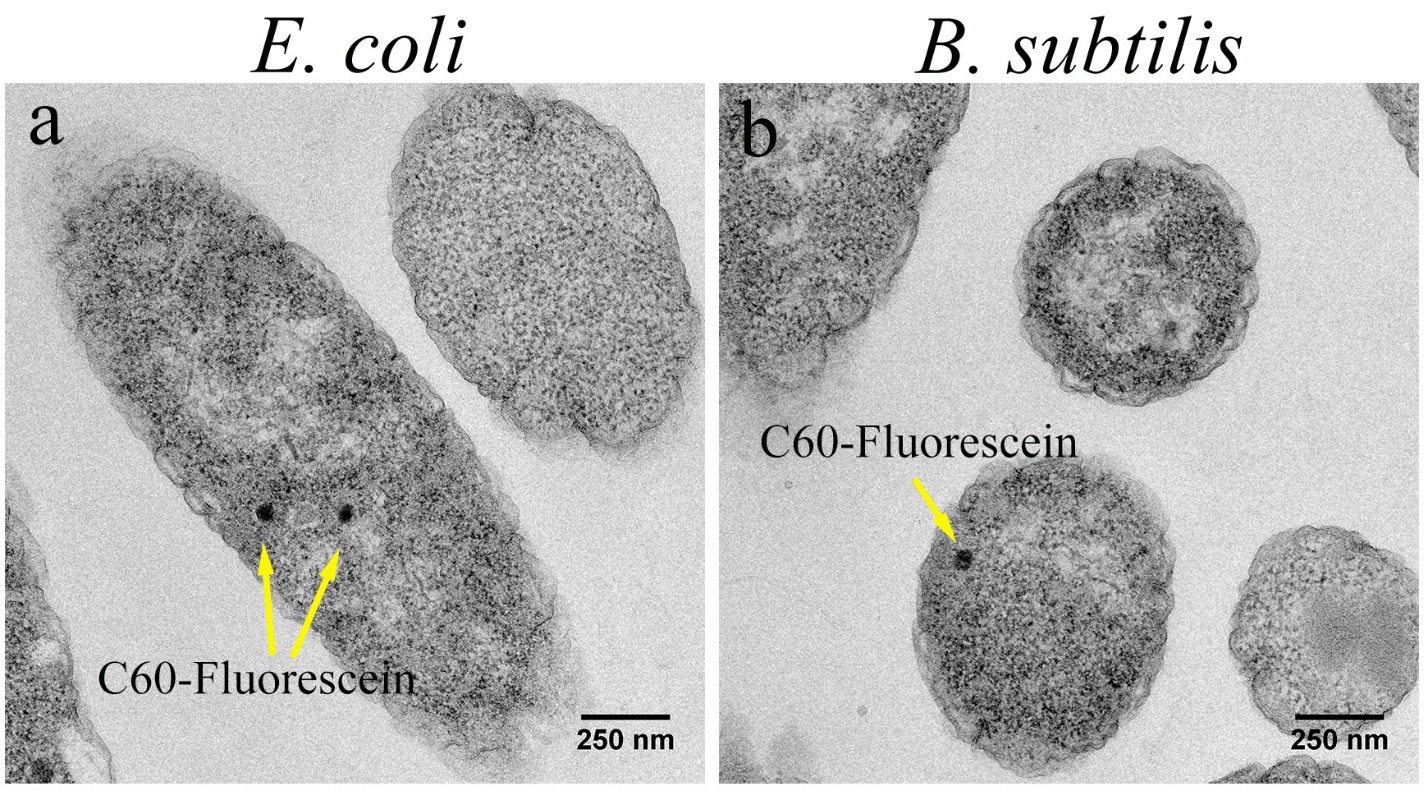


Additional Figure S2. Cellular uptake of C60-Fluorescein by *E. coli* and *B. subtilis* monitored by Transmission Electron Microscopy. The dark spots, pointed by the yellow arrows, represent C60-Fluorescein uptake by *E. coli* (a) and *B. subtilis* (b). Cellular uptake of C60 pyrrolidine tris-acid was reported in the previous publication [[1](#_ENREF_1)]. Overall, the results show that the functionalization of C60 pyrrolidine tris-acid does not affect diffusion of the C60 complex into bacteria cell body.


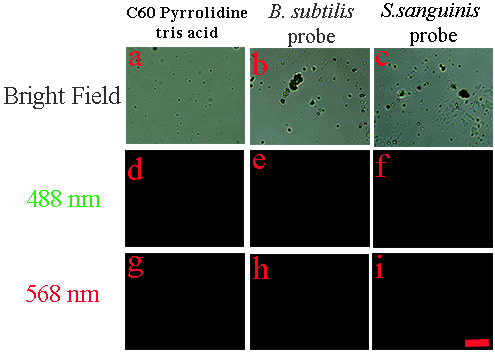


Additional Figure S3. Auto-fluorescence of the C60-rRNA detector-reporter complexes, synthesized for *B. subtilis* and *S. sanguinis* specific probes, is reported in the absence of the bacteria and monitored by fluorescent microscopy. (a-c) C60 pyrrrolidine tris acid, C60-rRNA detector-reporter complex synthesized for *B.subtilis, and* C60-rRNA detector-reporter complex synthesized for *S.sanguinis* monitored in bright field. (d-f) auto-fluorescence of fields of views from (a-c) monitored by the fluorescence microscopy at 488nm. (g-i) auto-fluorescence of fields of views from (a-c) monitored by fluorescence the microscopy at the 568nm excitation frequency. Scale bar is 10 µm.


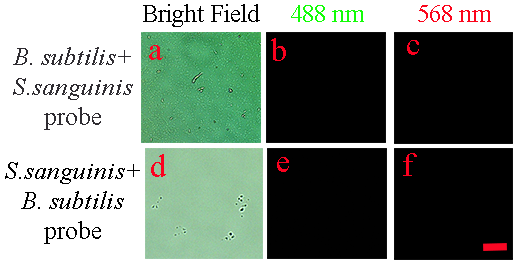


Additional Figure S4. Mismatch incubation of probe complexes with bacteria shows no hybridization monitored by fluorescent microscopy. (a) *S. sanguinis* probe complexes incubated with *B. subtilis* bacteria imaged in bright field. (b-c) *S. sanguinis* probe complexes incubated *B. subtilis* have no fluorescent signal under 488 or 568nm excitations frequencies. (d) *B. subtilis* probe *complexes* incubated with *S.* *sanguinis* and imaged in the bright field. (e-f) *B. subtilis* probe complexes incubated with *S. sanguinis* have no fluorescent signal under 488 or 568nm excitations frequencies. Scale bar is 10µm.

**Reference**

1. Cheng Q, Aravind A, Buckley M, Gifford A, Parvin B: **Functionalized Buckyballs for Visualizing Microbial Species in Different States and Environments**. *Scientific reports* 2015, **5**:13685.
